# ISGylation is disrupted by *UBA7* gene variants identified in individuals with neurodevelopmental disorder phenotypes

**DOI:** 10.1016/j.isci.2026.115454

**Published:** 2026-03-30

**Authors:** Venkateshwarlu Bandi, Myrrhe Venema, Iona Wallace, Merel O. Mol, Anita Nikoncuk, Rachel Schot, Marjon van Slegtenhorst, Emilia K. Bijlsma, Amjad Khan, Susan M. White, Rocio Rius, Martin B. Delatycki, Vinodh Narayanan, Kirby N. Swatek, Tahsin Stefan Barakat, Francisco Bustos

**Affiliations:** 1 Pediatrics and Rare Diseases Group, Sanford Research, Sioux Falls, SD 57104, USA; 2 Department of Clinical Genetics, Erasmus MC University Medical Center, 3000 CA Rotterdam, the Netherlands; 3 Discovery Unit, Department of Clinical Genetics, Erasmus MC University Medical Center, 3000 CA Rotterdam, the Netherlands; 4 MRC Protein Phosphorylation and Ubiquitylation Unit, School of Life Sciences, University of Dundee, Dundee DD1 5EH, UK; 5 Department of Clinical Genetics. LUMC University Medical Center, 2333 ZA Leiden, the Netherlands; 6 Department of Zoology, University of Lakki Marwat, Lakki Marwat 28420, Khyber Pakhtunkhwa, Pakistan; 7 Victorian Clinical Genetics Services, Murdoch Children’s Research Institute, Parkville, VIC 3052, Australia; 8 Department of Paediatrics, University of Melbourne, Parkville, VIC 3010, Australia; 9 Centre for Population Genomics, Garvan Institute of Medical Research and UNSW Sydney, Sydney, NSW 2010, Australia; 10 Centre for Population Genomics, Murdoch Children’s Research Institute, Melbourne, VIC 3052, Australia; 11 Bruce Lefroy Centre, Murdoch Children’s Research Institute, 50 Flemington Road, Parkville, VIC 3052, Australia; 12 Center for Rare Childhood Disorders (C4RCD), Translational Genomics Research Institute (TGen), Phoenix, AZ 85004, USA; 13 Department of Pediatrics, University of South Dakota, Sioux Falls, SD 57105, USA

**Keywords:** Genetics, Molecular biology, Neuroscience

## Abstract

ISGylation is a ubiquitin-like enzymatic cascade that transfers the small modifier ISG15 to lysine residues of protein substrates. ISGylation occurs in a three-step enzymatic cascade involving UBA7 (E1), UBE2L6 (E2), and HERC5, TRIM25, or human homolog of ariadne (HHARI) (E3) enzymes. This mechanism regulates core cellular processes, but its role in neurodevelopmental disorders remains unclear. Here, we identified individuals with neurodevelopmental disorder phenotypes harboring biallelic *UBA7* gene variants and assessed their functional effects. Truncating *UBA7* variants result in loss of catalytic activity, protein stability, and localization. In contrast, a missense variant drives no functional defects. Fibroblasts harboring the variant p.Lys709Serfs∗45 had reduced *UBA7* transcript and produced a truncated and unstable UBA7 protein. These fibroblasts were unable to induce ISGylation upon interferon beta treatment, indicating a dysfunctional ISGylation system. Together, our findings identify cellular mechanisms disrupted by *UBA7* variants and lay the foundation for uncovering the role of the ISGylation system and UBA7 in neurodevelopment.

## Highlights

• Individuals with neurodevelopmental disorder phenotypes harbor *UBA7* gene variants
• Two identified *UBA7* variants result in disrupted UBA7 enzymatic activity
• *UBA7*-variant fibroblasts express reduced *UBA7* mRNA and an unstable UBA7 protein
• *UBA7*-variant fibroblasts are devoid of interferon-induced ISGylation

## Graphical abstract

**Figure:**
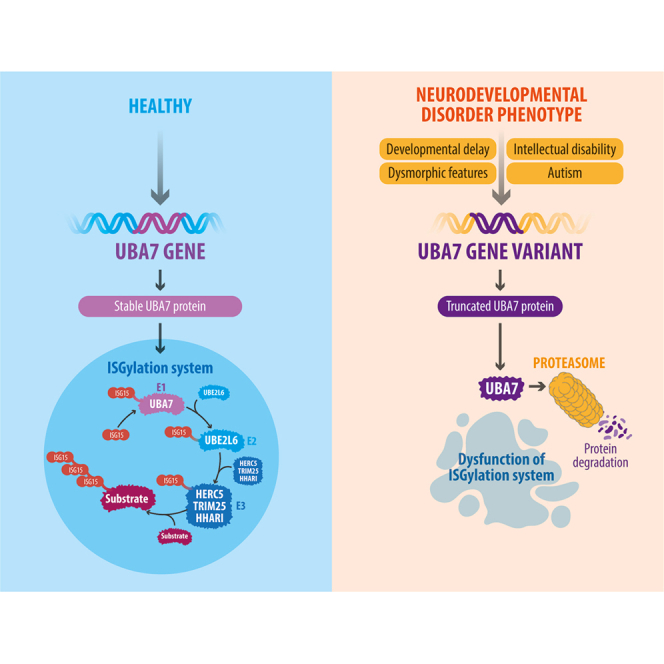

## Introduction

The posttranslational conjugation of the ubiquitin-like modifier interferon-stimulated gene 15 (ISG15) to protein substrates known as ISGylation is mediated by the sequential action of three enzymes.^1^ UBA7 (also known as UBE1L) is the E1 activating enzyme of the system that works in an ATP-dependent manner.^2^^,^^3^^,^^4^ Transthiolation reactions mediate the transfer of ISG15 to the E2 conjugating enzyme UBE2L6^5^ and to the ISG15-specific E3 ligase enzyme HECT and RCC1-containing protein 5 (HERC5), which is the major E3 involved in this process.^6^^,^^7^ Tripartite motif-containing protein 25 (TRIM25) and human homolog of ariadne (HHARI) E3 ligases have also been shown to function in this system.^8^^,^^9^ These enzymes transfer ISG15 to lysine residues of protein substrates, and this reaction is reversed by the activity of the ISG15-specific deubiquitylating enzyme USP18.^10^

ISG15 and the enzymatic components of the ISGylation pathway are transcriptionally induced in response to stimuli, including type I interferons (IFN-I), interleukins, lipopolysaccharides, retinoic acid, hypoxia, and DNA damage to modulate core biological processes.^11^^,^^12^ Consistent with this importance, the dysregulation of ISGylation is linked to human pathologies such as cancer,^13^ where ISGylation-related genes are either elevated or reduced depending on the cancer type.^11^^,^^13^ Inherited deficiency of components of the ISGylation system, such as ISG15 and USP18, has been associated with immune and degenerative conditions. *ISG15* deficiency is characterized by brain calcification, seizures, Mendelian susceptibility to mycobacteria, and dermal lesions.^14^^,^^15^^,^^16^^,^^17^ In contrast, *USP18* deficiency leads to perinatal death with a phenotype consistent with pseudo-TORCH syndrome characterized by excessive inflammation, brain calcification, and polymicrogyria.^18^^,^^19^^,^^20^ Individuals with these disorders have dysregulated IFN-I signaling that drives their phenotypes.^17^^,^^21^

Although *UBA7* has been suggested as an intellectual disability-associated gene in large-scale cohort studies,^22^^,^^23^ the functional significance of *UBA7* variants found in individuals with neurodevelopmental disorders has not been defined. Here, we identified three unrelated individuals presenting with global developmental delay, intellectual disability, autism, and unspecific dysmorphic features that harbored biallelic, protein-truncating *UBA7* variants and assessed the potential functional relevance of selected variants via biochemical assays. We found that the *UBA7* variants p.Trp311∗ and p.Lys709Serfs∗45 result in loss of UBA7 catalytic activity in biochemical assays. Fibroblasts from two individuals harboring the p.Lys709Serfs∗45 variant have reduced *UBA7* transcript, translate a truncated and unstable protein, and lack ISGylation activity. Together, our results indicate that *UBA7* variants disrupt UBA7 function, suggesting possible involvement of ISGylation in neurodevelopment.

## Results

### Identification and clinical assessment of individuals with *UBA7* variants

**Figure 1:**
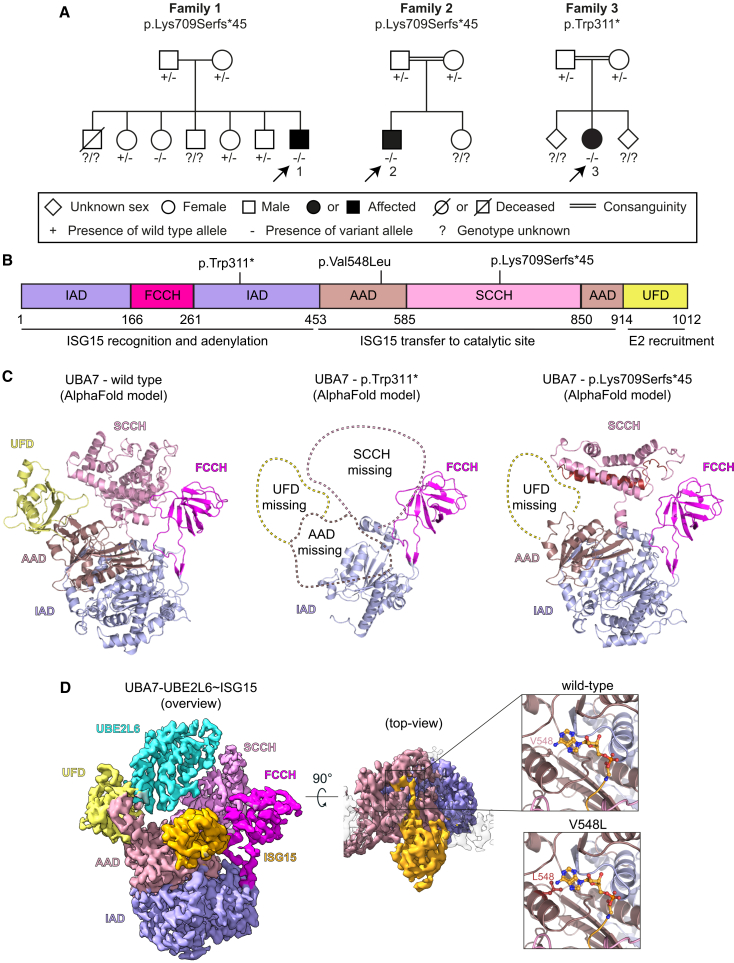
*UBA7* variants from individuals with neurodevelopmental disorders alter protein structure (A) Pedigrees of the families studied in this article indicate the affected individuals 1, 2, and 3. A legend is provided for the symbols used. (B) Diagram of UBA7 protein domains and their functions. The location of the identified variants in the structure is indicated. IAD: inactive adenylation domain, AAD: active adenylation domain, FCCH: first catalytic cysteine half-domain, SCCH: second catalytic cysteine half-domain, UFD: ubiquitin-fold domain. (C) Alpha-fold models of UBA7 protein resulting from *UBA7* wild type, or p.Trp311∗ and p.Lys709Serfs∗45 variants. The Red helix represents the added residues resulting from the p.Lys709Serfs∗45 frameshift variant. (D) Structure of wild-type UBA7 in complex with UBE2L6 and ISG15 (left; EMB-16891; pdb:8OIF). The top-view (middle) and zoomed areas (right) highlight the adenylation pocket and location of the p.Val548Leu mutation shown in red.

Following the initial identification of an individual with neurodevelopmental delay harboring a homozygous truncating variant in *UBA7*, we identified two additional individuals with homozygous truncating variants and one individual with a homozygous missense variant through international collaboration (Figures 1A and 1B; Table 1).

**Table 1:** Clinical overview of individuals with *UBA7* variants

General		Individual 1	Individual 2	Individual 3
*Sex*		male	male	female
*Ethnicity*		Libyan	Turkish	Pakistani
*Deceased*		no	no	no
*Anamnestic consanguinity in family*		no	yes	yes

**Genetic analysis**				

*Regions of homozygosity present*		yes, large ROH chr3q	yes	N/A
*Genomic position variant (hg38)*		chr3:g.49809426_49809429del	chr3:g.49809426_49809429del	chr3:g.49811876C>T
*Transcript variant (*NM_003335.3*)*		c.2126_2129del	c.2126_2129del	c.933G>A
*UBA7 Variant (protein)*		p.Lys709Serfs∗45	p.Lys709Serfs∗45	p.Trp311∗
*Zygosity*		homozygous	homozygous	homozygous
*Functional analysis variant*		loss of function	loss of function	loss of function

***GnomAD v4.1.0***				

*Allele number*		1614216	1614216	absent
*Allele count*		976	976	absent
*Allele frequency*		0.0006046	0.0006046	absent
*Number of homozygotes*		1	1	absent

***GnomAD v4.1.0 - non-UKB***				

*Allele number*		781106	781106	absent
*Allele count*		490	490	absent
*Allele frequency*		0.0006273	0.0006273	absent
*Number of homozygotes*		0	0	absent

**Variant predictions**				

*CADD (phred)*		24.2	24.2	36.0
*MutationTaster*		disease-causing, probability score 1	disease-causing, probability score 1	disease-causing, probability score 1
**HPO term**	**Clinical Phenotype**			
*HP:0001263*	*Developmental delay*	yes	yes	yes
*HP:0001270*	*Speech development*	delayed	delayed	normal
*HP:0000750*	*Motor development*	delayed	normal	delayed
*HP:0002376*	*Regression*	no	no	yes
*HP:0001249*	*Intellectual disability*	yes	yes	yes
	*IQ tested*	62	55	52
*HP:0000717*	*Autism*	yes	yes	yes
*HP:0001250*	*Epilepsy/seizures*	no	no	yes, focal
	*Responding to therapy*	–	–	yes, carbamazepine 250 mg once per day
*HP:0034435*	*Visual interaction*	very little	no	no
*HP:0008936*	*Axial hypotonia*	yes	no	yes
*HP:0001257*	*Spasticity*	no	no	N/A
*HP:0007141*	*Neuropathy*	no	N/A	N/A
	*MRI findings*	N/A	N/A	N/A
	D*ysmorphic features*	yes	yes	yes
*HP:0001999*	F*acial dysmorphisms*	brachycephaly, low frontal hairline, synophris, large earlobes, large incisors	no	microcephaly, high anterior headline, sparse eyebrows, hypertelorism, downslanting palpebral features, low-set ears, large earlobes, rounded nasal tip, flattened nasal bridge, thin upper lip, micrognathia, and gingival overgrowth
	O*ther dysmorphisms*	hypoplasia of toenails (fifth toe), acanthosis nigricans, café au lait macula, hairy arms and legs	Pes planus, gynaecomastia, café au lait macula	–
	O*ther*	obesity, eczema, premature adrenarche	dental problems (asymmetric occlusion, narrow maxilla, crossbite), eczema	recurrent infections, low white blood cell count

Individual 1 is a currently 12-year-old male from the Netherlands, of Libyan descent. He was born to non-consanguineous parents after an uneventful pregnancy at 32 + 4 weeks. He comes from a family with 7 children, of which 6 are alive, and has 5 unaffected siblings. The family history was unremarkable for neurodevelopmental disorders. He presented at the department of Clinical Genetics at age 9 years, with a history of global developmental delay, with first words at age 5 years, and slightly delayed motor development. He made very little visual interaction. Intellectual disability was later confirmed with an IQ of 62. Furthermore, he showed signs of autism and behavioral concerns, including temper, skin picking, hyperphagia, and mild sleep disturbances. Apart from axial hypotonia, no focal neurological symptoms were noted. At age 10 years, his length measured 164.3 cm (SD 1.55), with a weight of 78 kg (SD 3.55) and a head circumference of 55.5 cm (SD 0.89). Further clinical findings included mild eczema and premature adrenarche. Dysmorphic features included brachycephaly and café au lait maculae, further summarized in Table 1.

Diagnostic testing included targeted analysis for Fragile X and Prader-Willi syndrome, a DNA-methylation Episign, and metabolic screening in plasma and urine, which were all unremarkable. The SNP array did not identify any relevant copy number variations (CNVs). Trio exome sequencing identified a homozygous truncating variant in the *UBA7* gene located on chromosome 3p21.31 (NM_003335.3:c.2126_2129del; p.Lys709Serfs∗45), of which both parents were heterozygous carriers (Figures 1A and 1B). This variant is found 490 times in a heterozygous but not homozygous state among the 781,106 alleles in the gnomAD v4.1.0 non-UKB cohort, and predicted *in silico* to be potentially damaging (Table 1). No other likely disease explaining variant was identified (Table S1). Although initially unavailable, ultimately four out of five living siblings were available for segregation analysis. Of these four siblings, three were heterozygous carriers of the p.Lys709Serfs∗45 variant; however, in one sister, the variant was also present in a homozygous state. Although no detailed medical data are available, she was reported to have a normal development without neurological symptoms.

Individual 2 is a currently 23-year-old male from the Netherlands, of Turkish descent. He was born at term after an uneventful pregnancy to consanguineous, unaffected parents (first cousins). He has one older sister who follows special education, but otherwise has no noteworthy medical history, and who could not be further investigated. He first presented at the department of Pediatrics at age of 5 years because of developmental delay of speech with normal motor development and autism. Intellectual disability was confirmed with an IQ of 55. He made no visual interaction. He had sleeping problems at a younger age, though these resolved later in life. No other focal neurological symptoms were noted.

At age 16 years, his length measured 176.5 cm (SD -0.44), with a weight of 84.2 kg (weight-length SD 2.36). Further clinical findings included eczema and dental abnormalities, including asymmetric occlusion, narrow maxilla, and cross bite. There were no facial dysmorphisms, but other features noted during examination were pes planus, gynaecomastia, and two café au lait maculae.

Metabolic diseases screening in urine was unremarkable. Trio exome sequencing revealed the same homozygous truncating variant in *UBA7* (NM_003335.3:c.2126_2129del; p.Lys709Serfs∗45) as found in individual 1 (Figures 1A and 1B; Table 1), with no other likely disease-causing variant identified (Table S1).

Individual 3 is a currently 22-year-old female from Pakistan. She was born to first-cousin parents at 38 weeks after a pregnancy complicated by intra-uterine growth retardation, and has two unaffected siblings who could not be investigated. She presented at 6 years of age with global developmental delay, later developing into moderate intellectual disability, early-onset seizures, and repetitive behaviors. Her speech development was normal, with her first words spoken at 16 months of age. Her motor development, however, was slightly delayed, with independent sitting at 16 months and independent walking at 20 months. Other neurological symptoms included axial hypotonia and mild dystonia. She made no visual interaction. She experienced focal epileptic seizures, which responded well to carbamazepine 250 mg once daily. Further clinical findings include recurrent infections with a low white blood cell count. Dysmorphic features are summarized in Table 1.

Trio exome sequencing revealed a homozygous truncating variant in *UBA7* (NM_003335.3:c.933G>A; p.Trp311∗), of which both parents were heterozygous carriers (Figures 1A and 1B). The variant is absent in gnomAD and *in silico* predicted to be possibly disease-causing (Table 1).

Finally, we identified a fourth individual, harboring a homozygous NM_003335.3:c.1642G>T; p.Val548Leu variant of uncertain significance in *UBA7*. Most *in silico* tools predicted a benign effect of this variant, and the clinical phenotype of this individual is most likely explained by a homozygous missense variant of uncertain significance in *RFT1*, causing a congenital disorder of glycosylation syndrome (CDG) (Figure S1; Table S2; Data S1). We therefore consider the *UBA7* variant p.Val548Leu as likely benign and not disease contributing, and included this variant as a negative control in our functional experiments.

### Protein truncating *UBA7* variant disrupts UBA7 enzymatic activity

To gain insight into the potential impact of the identified *UBA7* variants on protein biology, we first predicted the structure of UBA7 with the p.Trp311∗ and p.Lys709Serfs∗45 variants using AlphaFold. We observed that the p.Trp311∗ variant is predicted to generate a truncated protein that lacks sections of the inactive adenylation domain (IAD) and the entire active adenylation domain (AAD), second catalytic cysteine half domain (SCCH), and ubiquitin-fold domain (UFD), which together control most activities of this enzyme.^2^^,^^3^ The p.Lys709Serfs∗45 variant is predicted to generate a truncated protein lacking sections of the SCCH and AAD domains, and the entire UFD domain, which is critical for coordinating the E2 enzyme (Figure 1C). To assess the potential impact of the p.Val548Leu variant on UBA7 structure, we interrogated our published structure of UBA7 in a complex with UBE2L6 and ISG15 (pdb:8OIF).^3^ Valine 548 is located near the UBA7 adenylation pocket. When we replaced this residue with leucine *in silico*, no major steric clashes were observed in the structure (Figure 1D), consistent with variant pathogenicity prediction tools (Table S2).

**Figure 2:**
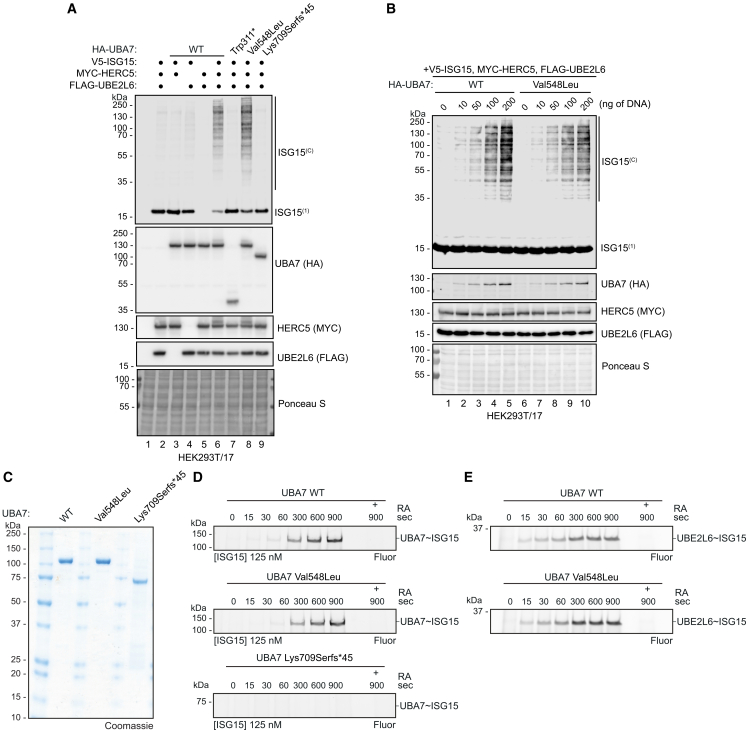
Protein-truncating *UBA7* variants alter catalytic activity (A) Protein truncating *UBA7* variants p.Trp311∗ and p.Lys709Serfs∗45 lack ISGylation activity in cells. Protein ISGylation assay in HEK293T/17 cells transfected with the indicated vectors. Formation of ISG15 conjugates (ISG15^(C)^) and the presence of free ISG15 (ISG15^(1)^) was analyzed via V5-tag immunoblotting. Transfected UBA7 (HA), HERC5 (Myc), and UBE2L6 (FLAG) protein levels were also analyzed (*n* = 3). (B) A *UBA7* p.Val548Leu variant does not affect UBA7 activity. Protein ISGylation assay in HEK293T/17 cells transfected with the indicated vectors and analyzed as in A. Ponceau staining of the membranes in A and B is shown as a loading control (*n* = 2). (C) Purified UBA7 wild type and UBA7 mutants visualized by Coomassie-stained SDS-PAGE gels (*n* = 1). (D) UBA7 charging reactions with UBA7 wild type (WT), UBA7 Val548Leu and UBA7 Lys709Serfs∗45. Reactions were separated by SDS-PAGE and visualized with fluorescent imaging (Fluor) for the indicated times in seconds (sec) (*n* = 3). (E) Comparison of UBA7 wild-type- and UBA7 Val548Leu-mediated UBE2L6 charging with ISG15. Reactions were separated by SDS-PAGE and visualized with fluorescent imaging (*n* = 3). As a control for UBA7∼ISG15 or UBE2L6∼ISG15 thioester formation, a 900-s reaction was mixed with LDS sample buffer containing reducing agent (RA), as shown in D and E.

To address the impact of *UBA7* variants on ISGylation activity, we reconstituted the ISGylation system in HEK293T/17 cells as a functional assay.^1^^,^^2^ In these assays, we assessed the *UBA7* variants p.Lys709Serfs∗45 (found in two individuals) and p.Trp311∗, and used the likely benign *UBA7* variant p.Val548Leu as a control. When we combined wild-type HA-UBA7 with V5-ISG15, Myc-HERC5, and FLAG-UBE2L6, we observed efficient generation of V5-ISG15 conjugates, which is reduced by the lack of any of the components (Figure 2A, compare lanes 1–5 to lane 6). Use of HA-tagged UBA7 Trp311∗ or Lys709Serfs∗45, which have a lower molecular weight as expected, in this assay, drastically reduced ISGylation (Figure 2A, compare lanes 7 and 9 to lane 6). Contrary to HA-tagged UBA7 Val548Leu was as effective as the wild type in inducing ISGylation in this assay (Figure 2A, compare lane 8 to lane 6) at any DNA concentration tested (Figure 2B, compare lanes 6–10 to 1–5).

To directly test the impact of these variants on the core catalytic mechanisms of UBA7, we purified recombinant wild-type and mutant UBA7 (Figure 2C) and subjected these proteins to E1 charging reactions using fluorescently labeled ISG15. We observed that UBA7 wild type and UBA7 Val548Leu are efficiently charged with ISG15 in a time course, but no charging was observed when UBA7 Lys709Serfs∗45 was used in the assay during the same period (Figure 2D). This indicates that the Lys709Serfs∗45 variant impairs the ability of UBA7 to be loaded with ISG15, which is a key step in the ISGylation cascade. To assess if the Val548Leu variant affected the ISG15 E1-E2 transthiolation reaction, we subjected recombinant wild-type or Val548Leu UBA7 to a UBE2L6 E2 charging assay using fluorescent ISG15. No differences were observed in the ability of UBA7 Val548Leu to transfer ISG15 to the E2 in this assay (Figure 2E). UBA7 Trp311∗ was not included in these analyses because it lacks the domains required for ISG15 recognition, activation, and transfer to the E2 enzyme (Figure 1C).^2^^,^^3^ Together, these data demonstrate that the p.Lys709Serfs∗45 but not the p.Val548Leu variant disrupts UBA7 catalytic activity *in vitro*.

### Protein-truncating *UBA7* variants alter protein stability and localization

**Figure 3:**
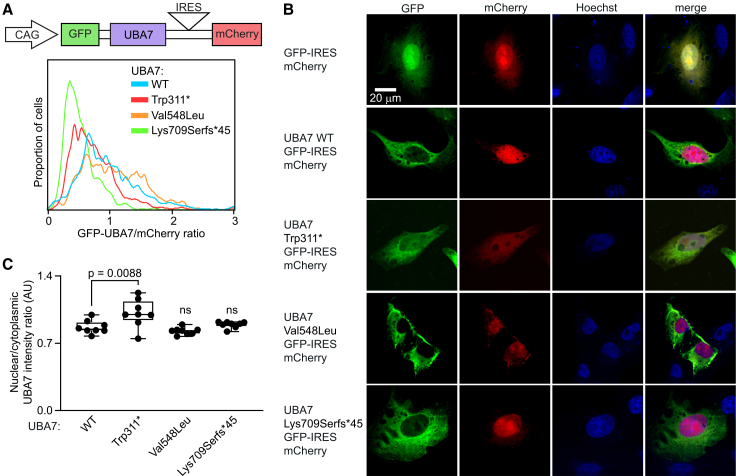
Protein-truncating *UBA7* variants alter protein stability and subcellular localization (A) Top: diagram of the reporter construct used, which contains GFP-tagged UBA7, an IRES sequence and mCherry controlled by an upstream CAG promoter. Bottom. Flow cytometry histogram representation of a reporter assay performed in HEK293T/17 cells transfected with GFP-IRES-mCherry plasmids encoding *UBA7* wild type (WT), p.Trp311∗, Val548Leu, and Lys709Serfs∗45 vectors (*n* = 3). See also Figure S2. (B) Super-resolution confocal images of U-2 OS cells transfected with the indicated bicistronic vectors. GFP and mCherry fluorescent signals were imaged, and Hoechst staining was used to mark the DNA. Scalebar: 20 μm. (C) Quantification of the nuclear to cytoplasm GFP-UBA7 intensity ratios from B. Data are presented as a Box-and-Whiskers plot indicating minimum and maximum values, and the median. One-way ANOVA followed by Tukey’s post-hoc test statistical differences are shown as not significant (ns) or the *p*-value. Images were obtained and quantified from all cells present in 8 fields per condition.

We next tested if these *UBA7* variants had an impact on other aspects of UBA7 protein biology, such as protein stability and subcellular localization. For this, we created a bicistronic construct encoding GFP-tagged wild-type or mutant UBA7, which are translated in a CAP-dependent manner, followed by an internal ribosome entry sequence (IRES), which drives the translation of mCherry as an internal control. Using these constructs, we performed a protein stability reporter assay via flow cytometry^24^ that we have previously used to assess the impact of disease-associated gene variants.^25^ In this assay, we found that UBA7 Trp311∗ and Lys709Serfs∗45 but not Val548Leu show lower protein stability in HEK293T/17 cells as observed when quantifying the GFP-UBA7 to mCherry ratios (Figures 3A and S2). We then used these constructs to describe the localization of wild-type or mutant UBA7 in U-2 OS cells using confocal microscopy. Wild-type GFP-tagged UBA7 is mainly a cytoplasmic protein, with some nuclear localization, which is also observed for GFP-tagged UBA7 Val548Leu and Lys709Serfs∗45 (Figures 3B and 3C). However, UBA7 Trp311∗ displayed increased nuclear localization (Figures 3B and 3C). Together, these data indicate that the *UBA7* variants p.Lys709Serfs∗45 and p.Trp311∗ impair protein stability and that *UBA7* p.Trp311∗ results in increased UBA7 nuclear localization.

### Patient fibroblasts with a homozygous p.Lys709Serfs∗45 *UBA7* variant have reduced *UBA7* transcript and produce an unstable UBA7 protein

**Figure 4:**
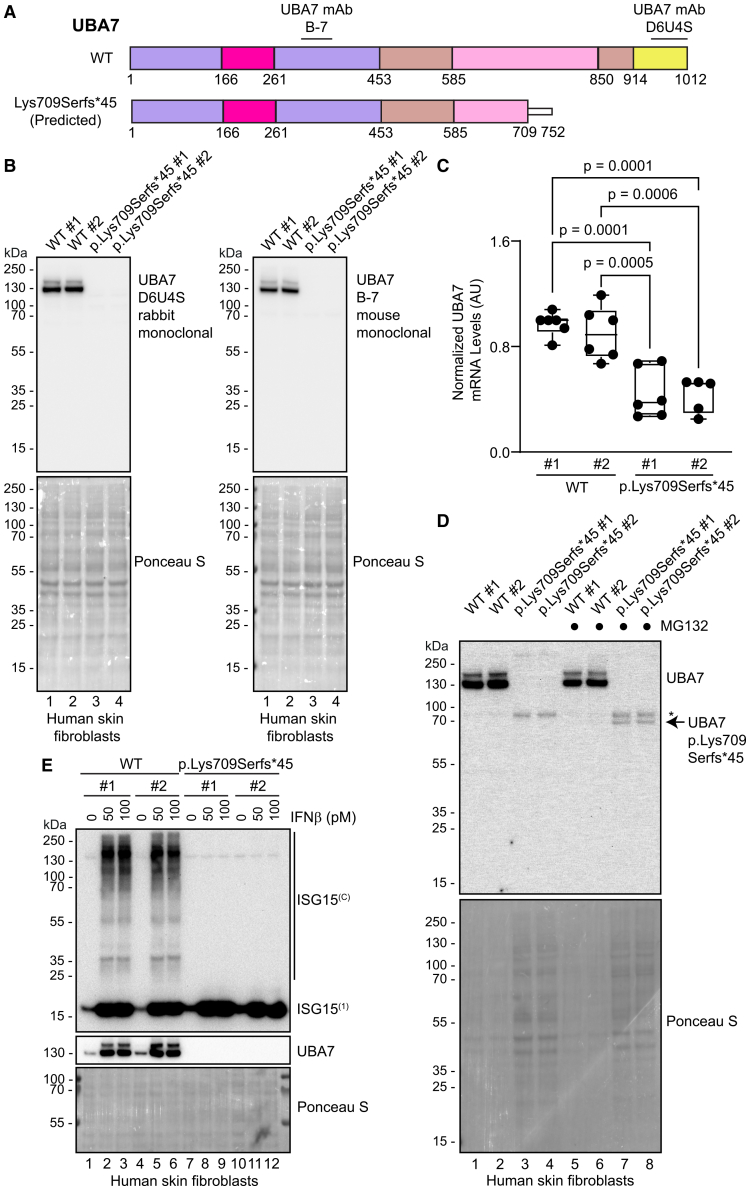
Human skin fibroblasts with a *UBA7* p.Lys709Serfs∗45 variant lack UBA7 expression and functional ISGylation (A) Diagram of the UBA7 protein domain structure comparing wild type to the predicted truncated protein generated by the p.Lys709Serfs∗45 variant. The location of the peptides used to raise the indicated monoclonal antibodies (mAb) is shown. (B) UBA7 protein expression of human skin fibroblasts from two individuals carrying the *UBA7* p.Lys709Serfs∗45 variant or two wild-type (WT) control individuals was analyzed via immunoblotting using the indicated antibodies (*n* = 3). Ponceau S staining is shown as a loading control. (C) *UBA7* transcript levels of two fibroblast lines carrying the *UBA7* p.Lys709Serfs∗45 variant and two wild-type control (WT) lines were analyzed via quantitative RT-PCR (*n* = 6). Data are presented as a Box-and-Whiskers plot indicating minimum and maximum values, and the median. One-way ANOVA followed by Tukey’s post-hoc test *p*-values are indicated. (D) Fibroblasts with a UBA7 p.Lys709Serfs∗45 variant express an unstable truncated UBA7 protein (arrow). The indicated cell lines were treated with the proteasome inhibitor MG132 or DMSO as a control, and UBA7 levels were analyzed via immunoblotting. Twice the amount of protein was intentionally loaded for the mutant samples compared to the control samples in this experiment (*n* = 3). Asterisk indicates a non-specific band. Ponceau staining is shown as a loading control. (E) Generation of ISGylation conjugates (ISG15^(C)^) in response to 0 (control), 50, or 100 pM interferon beta (IFNβ) treatment in control or p.Lys709Serfs∗45 fibroblast lines was analyzed via ISG15 immunoblotting. UBA7 was analyzed to confirm cell line identity, and Ponceau S staining is shown as a loading control (*n* = 3).

To better understand the cellular impact caused by *UBA7* variants in humans, we obtained skin fibroblasts from individuals 1 and 2, harboring the homozygous p.Lys709Serfs∗45 variant, and compared them to fibroblasts from two healthy donors. Fibroblasts from individuals 3 and 4 were not available. Human skin fibroblasts express basal levels of UBA7 protein.^26^ We first compared the endogenous UBA7 protein levels in control or *UBA7* p.Lys709Serfs∗45 fibroblasts using two different monoclonal antibodies that were raised against a region toward the amino terminus (B-7 clone) or against the carboxy terminus of UBA7 (D6U4S clone) (Figure 4A). When we analyzed the UBA7 protein levels using immunoblotting, we detected no UBA7 protein in the two fibroblast lines carrying the *UBA7* p.Lys709Serfs∗45 variant, despite a strong and consistent detection of the protein in the two independent control cell lines using either antibody (Figure 4B, compare lanes 3–4 to 1–2). This lack of protein expression could be a consequence of changes in either mRNA expression or protein stability. We analyzed endogenous *UBA7* mRNA levels of these cell lines using quantitative RT-PCR. We observed that *UBA7* p.Lys709Serfs∗45 mutant fibroblast cell lines showed a significant decrease in *UBA7* transcript (Figure 4C), indicating that this may in part explain the lack of UBA7 protein expression in these cells. We next assessed if endogenous UBA7 protein stability was affected by the *UBA7* p.Lys709Serfs∗45 variant, as observed when analyzing GFP-tagged protein (Figure 3A). When we treated wild-type or *UBA7* mutant cells with the proteasome inhibitor MG132, we detected a low-intensity band in the expected size for the predicted truncated UBA7 protein via immunoblot (Figure 4D, compare lanes 7–8 to 3–4). Together, these results indicate that the *UBA7* p.Lys709Serfs∗45 variant drives a decrease in *UBA7* transcript levels and that the remaining translated protein is unstable and rapidly degraded by the proteasome.

### Patient fibroblasts with a homozygous p.Lys709Serfs∗45 *UBA7* variant lack ISGylation activity

Finally, we assessed the impact of the *UBA7* p.Lys709Serfs∗45 variant on the physiological function of UBA7. When we treated control fibroblast cells with interferon beta for 48 h, we observed the efficient formation of endogenous ISG15 conjugates along with the induction of UBA7 and ISG15, as expected, since these products are known interferon-inducible genes^27^^,^^28^ (Figure 4E, compare lanes 2–3 to 1, and 5–6 to 4). Although *UBA7* p.Lys709Serfs∗45 mutant fibroblasts could induce free ISG15 in response to the treatment, no ISG15 conjugates were detected (Figure 4E, compare lanes 8–9 to 2–3 and 11–12 to 5–6), indicating that cells carrying the homozygous *UBA7* p.Lys709Serfs∗45 variant lack ISGylation activity in response to interferon beta.

## Discussion

Here we describe two biallelic protein-truncating UBA7 gene variants found in three individuals presenting with developmental delay, intellectual disability, autistic behavior, and non-specific dysmorphic features. *In silico* protein modeling and functional studies provide evidence of UBA7 dysfunction due to these variants, potentially implicating a role of UBA7 and the ISGylation pathway in neurodevelopment, which requires further investigation.

The *UBA7* p.Trp311∗ variant lacks most functional domains of this enzyme, which explains why it results in loss of UBA7 activity. The increased nuclear localization of UBA7 Trp311∗ likely results from the small size of the truncated protein, enabling diffusion through the nuclear envelope.^29^ The *UBA7* p.Lys709Serfs∗45 variant results in a truncated protein lacking the UFD domain, which mediates E2 interaction and therefore lacks a key feature of the enzyme’s activity. We found that this variant disrupted mRNA levels likely via nonsense-mediated decay. Additionally, the little remaining UBA7 Lys709Serfs∗45 protein synthesized in patient fibroblasts is unstable and can only be observed when proteasomal degradation is blocked. Both variants resulted in decreased UBA7 protein stability in flow cytometry-based reporter assays, indicating that proper integrity and folding of UBA7 domains are required for maintaining UBA7 stability. Despite our observations on the stability and localization of the *UBA7* p.Trp311∗ variant, the availability or engineering of cell lines harboring this variant will clarify whether it affects mRNA expression and if the truncated protein is expressed, and may drive any deleterious effects in the nucleus.

Population data together with our results highlight the need to clarify the pathogenicity and penetrance of the *UBA7* p.Lys709Serfs∗45 variant. It is noteworthy that the p.Lys709Serfs∗45 variant is once present in a homozygous state in a male in gnomAD v4.1.0 when the UK Biobank cohort is included. When diagnostic analysis of individuals 1 and 2 was initially performed, older versions of gnomAD were used, in which there were no homozygotes for this variant, triggering the functional studies described herein to clarify the role of *UBA7* as a potential neurodevelopmental disorder candidate gene. The UK Biobank has no phenotypic exclusion, which might lead to more homozygotes for recessive conditions in gnomAD v4.^30^ Another explanation could be that the spectrum of consequences of the *UBA7* p.Lys709Serfs∗45 variant might be variable or with reduced penetrance, leading to the inclusion of homozygotes with a potentially milder phenotype. This could also be an explanation why the sister of individual 1, harboring the same homozygous p.Lys709Serfs∗45 variant, showed no clear phenotype. In the future, the availability of larger cohorts and pedigrees of patients carrying *UBA7* variants will clarify the phenotypical spectrum associated with these variants.

Because ISGylation has been extensively studied in the context of antiviral response and IFN-I signaling, this raises the question of whether *UBA7* variants may drive the observed phenotypes through defects in IFN-I signaling. Elevated IFN-I signaling is known to drive severe neurodevelopmental disorders such as Aicardi-Goutières syndrome, which is associated with variants in multiple IFN-I signaling regulators.^31^^,^^32^ Components of the ISGylation pathway regulate IFN-I signaling via a mechanism in which ISG15 stabilizes USP18, thereby negatively regulating IFN-I-induced responses.^21^^,^^28^^,^^33^ Consistently, syndromes associated with ISG15 or USP18 have been classified as interferonopathies due to their excessive IFN-I signatures.^34^ However, the role of UBA7 in regulating IFN-I signaling is still emerging,^35^ and current evidence suggests that UBA7 is dispensable for ISG15-USP18-mediated fine-tuning of IFN-I signaling in humans.^21^^,^^33^

The main clinical manifestations of individuals with truncating *UBA7* variants described here are developmental delays and intellectual disability, autism, hypotonia, and seizures. No magnetic resonance imaging was available, leaving it unclear whether these variants may also lead to brain calcifications or any other brain abnormalities. While individual 3 was reported to have recurrent infections and a low white blood cell count, this did not trigger further clinical immunological studies. It is therefore unclear whether and in which way immunological processes might be disrupted in these individuals. Targeted clinical studies testing these parameters in patients with *UBA7* variants, together with the elucidation of the role of UBA7 in regulating IFN-I signaling, may clarify if *UBA7* disruption conditions share clinical presentations with other ISGylation pathway-associated syndromes.

The identification of inactivating *UBA7* variants in individuals with neurodevelopmental disorder phenotypes, both in this study and previous reports,^22^^,^^23^ suggests that UBA7 may be involved in aspects of nervous system development. Our findings raise the question of whether UBA7 or ISGylation may play a role in neural cell differentiation, similar to their function in regulating hematopoietic stem cell cycle progression or leukemia cell differentiation.^36^^,^^37^ However, this possibility is not supported by *Uba7* knockout mouse models, which demonstrate that UBA7 is not essential for development, viability, or fertility in mice.^38^ This conflicting evidence may be explained by the evolutionary divergence in the ISGylation pathway.^12^^,^^21^ For example, murine ISG15 acts as an antiviral agent for a variety of viruses, whereas humans with *ISG15* deficiency are not susceptible to viral infection.^21^ These interspecies differences arise from the distinct regulation of IFN-I signaling: Human ISG15 negatively regulates IFN-I-induced responses by stabilizing USP18, while mouse ISG15 does not have a critical role in the USP18-mediated suppression of IFN-I signaling.^21^ Poor protein sequence identity among ISGylation system components further accounts for these differences.^33^ Therefore, defining UBA7’s role in neurodevelopment will require the development of human-based preclinical models. Future studies uncovering the specific stages and processes in which UBA7 is required during human nervous system development, together with the molecular defects and phenotypes resulting from additional *UBA7* patient variants from larger cohorts, will clarify if the ISGylation defect that we observe contributes to neurodevelopmental disorder pathogenesis.

### Limitations of the study

Although this study provides important insights into the cellular effects driven by *UBA7* variants found in individuals with neurodevelopmental disorders, several limitations warrant consideration. The small patient cohort and number of identified *UBA7* variants limit our conclusions on phenotypic variability and penetrance. Phenotypic characterization mostly relied on medical history, with no or limited systematic assessment of aspects such as immunity, brain structure and function, or access to tissue samples. The small cohort size also prevents us from determining any sex-related influences or associations. While *in silico*, *in vitro*, and in cell models helped assess variant impact on enzymatic activity, protein stability, and localization, these results cannot be directly extrapolated to what occurs in the affected individual’s cells. Further studies are needed to clarify the cellular consequences of the *UBA7* p.Trp311∗ variant on endogenous mRNA and protein expression and function. Finally, a major limitation of the study is that it does not provide a functional link between ISGylation dysfunction and clinical phenotypes. Future studies with larger patient cohorts and multidisciplinary mechanistic studies defining UBA7’s role in human neurodevelopment will be required to establish this link.

## Resource availability

### Lead contact

Requests for further information and resources should be directed to and will be fulfilled by the lead contact, Francisco Bustos (francisco.bustos@sanfordhealth.org).

### Materials availability

Mammalian expression plasmids generated in this study are available from Addgene https://www.addgene.org/Francisco_Bustos/ or directly from the lead contact. Plasmids in the pLIB backbone are available from Kirby Swatek (KSwatek001@dundee.ac.uk).

### Data and code availability

• All clinical data are presented herein. All data generated or analyzed during this study are included in this published article, except raw sequencing data, which, due to privacy regulations and given consent, cannot be publicly made available. Variants have been submitted to ClinVar.
• Original immunoblot, gel, and microscopy images for this study have been deposited at Mendeley Data and are publicly available as of the date of publication at https://doi.org/10.17632/xpbhrtx7nd.1.
• Any additional information required to reanalyze the data reported in this paper is available from the lead contact upon request.

## Acknowledgments

The authors thank the families who participated in this study. We thank Drs. Halim Loukil, Ikuo Masuho, and Kyle Roux (Sanford Research) for facilitating the obtention of control human fibroblasts and for HEK293T/17 and U-2 OS cells. We thank Dr. Michaela U. Gack (Florida Research and Innovation Center) for UBA7, HERC5, UBE2L6, and ISG15 plasmids. The Barakat lab was supported by the 10.13039/501100003246Netherlands Organisation for Scientific Research (ZonMw Vidi, grant 09150172110002). The Swatek lab is supported by the 10.13039/501100000265Medical Research Council (MC_UU_00038/8 and MC_UU_00018/10) and K.N.S. is a Lister Institute Prize Fellow. The Rare Disease Flagship acknowledges financial support from the 10.13039/100014607Royal Children's Hospital Foundation, the 10.13039/100014555Murdoch Children's Research Institute, Paula Fox, The Andrew and Geraldine Buxton Foundation and the 10.13039/501100022893Pierce Armstrong Foundation. Molecular graphics and analyses performed with UCSF ChimeraX, developed by the Resource for Biocomputing, Visualization, and Informatics at the University of California, San Francisco, with support from National Institutes of Health R01-GM129325 and the Office of Cyber Infrastructure and Computational Biology, National Institute of Allergy and Infectious Diseases. Funding bodies did not have any influence on study design, results, data interpretation, or the final manuscript.

## Author contributions

Conceptualization: T.S.B., F.B., and K.N.S.; data curation: V.B., M.V., M.O.M., A.N., I.W., M.v.S., E.K.B., A.K., S.M.W., R.R., M.B.D., V.N., and K.N.S.; formal analysis: V.B., M.V., M.O.M., I.W., K.N.S., and R.S.; investigation: V.B., M.V., M.O.M., I.W., and K.N.S.; supervision: T.S.B, F.B, and K.N.S.; visualization: V.B., F.B., I.W., K.N.S., and M.V.; writing – original draft: F.B. and M.V.; writing – review and editing: T.S.B, F.B, and K.N.S.

## Declaration of interests

Authors declare that they have no competing commercial interests.

## STAR★Methods

### Key resources table

**Table:** 

REAGENT or RESOURCE	SOURCE	IDENTIFIER
**Antibodies**		

rabbit monoclonal UBE1L/UBA7 (D6U4S), 1:1000	Cell Signaling Technology	Cat# 61266; RRID:AB_2799605
mouse monoclonal UBE1L (B-7), 1:2000	Santa Cruz Biotechnology	Cat# sc-390097
rabbit polyclonal ISG15, 1:4000	Proteintech	Cat# 15981-1-AP; RRID:AB_2126302
rabbit monoclonal V5-tag (D3H8Q), 1:1000	Cell Signaling Technology	Cat# 13202S; RRID:AB_2687461
Direct-Blot HRP anti-DYKDDDDK tag, 1:5000	Biolegend	Cat# 637311; RRID:AB_2566706
Direct-Blot HRP anti-HA.11 epitope tag, 1:5000	Biolegend	Cat# 901519; RRID:AB_2686981
HRP-Conjugated Myc-Tag (9B11), 1:5000	Cell Signaling Technology	Cat# 2040S; RRID:AB_2148465
mouse monoclonal beta Actin, 1:5000	Santa Cruz Biotechnology	Cat# sc-47778; RRID:AB_626632
Anti-mouse IgG, HRP-linked Antibody, 1:10,000	Cell Signaling Technology	Cat# 7076; RRID:AB_330924
Anti-rabbit IgG, HRP-linked Antibody, 1:10,000	Cell Signaling Technology	Cat# 7074; RRID:AB_2099233
IRDye 680LT Donkey anti-Mouse IgG, 1:10,000	LICORbio	Cat# 92668022; RRID:AB_10715072
IRDye 800CW Donkey anti-Rabbit IgG, 1:10,000	LICORbio	Cat# 92632213; RRID:AB_621848

**Bacterial and virus strains**		

DH10EMBacY E.Coli	Geneva Biotech	N/A
Stellar Competent Cells (*E. coli* HST08)	Takara Bio	Cat# 636763
NEB 5-alpha Competent E. coli (DH5α)	New England Biolabs	Cat# C2987
BL21-CodonPlus (DE3)-RIPL Competent Cells (*E. coli*)	Agilent Technologies	Cat# 230280

**Chemicals, peptides, and recombinant proteins**		

Interferon beta	Thermo Fisher Scientific	Cat# 300-02BC
MG132	MilliporeSigma	Cat# 474790
PEI MAX Linear Polyethylenimine Hydrochloride	Polysciences	Cat# 24765
Lipofectamine LTX Reagent with PLUS	Thermo Fisher Scientific	Cat# 15338100
BODIPY-labelled human ISG15 C0 C78S	Wallace et al.^3^	N/A
Human UBE2L6 protein	Wallace et al.^3^	N/A
Human UBA7 protein	Wallace et al.^3^	N/A
Human UBA7 p.Val548Leu protein	This paper	N/A
Human UBA7 p.Lys709Serfs∗45 protein	This paper	N/A

**Critical commercial assays**		

qScript cDNA Synthesis Kit	Quanta Bio	Cat# 95047-100
TB Green Premix Ex Taq	Takara Bio	Cat# RR820L
In-Fusion Master Mix	Takara Bio	Cat# 638947
Q5 High-Fidelity 2X Master Mix	New England Biolabs	Cat# M0492L
Pierce BCA Protein assay kit	Thermo Fisher Scientific	Cat# 23225

**Deposited data**		

UBA7 model	AlphaFold 2 Protein Structure Database	UniProt: P41226
UBA7-ISG15-UBE2L6 cryo-EM structure	Protein DataBank	PDB: 8OIF
*UBA7* p.Trp311∗ variant	ClinVar	VCV004540576.1
*UBA7* p.Val548Leu variant	ClinVar	VCV002351006.3
*UBA7* p.Lys709Serfs∗45 variant	ClinVar	VCV004540575.1
Original immunoblot images	This paper	Mendeley data https://doi.org/10.17632/xpbhrtx7nd.1
Original gel images	This paper	Mendeley data https://doi.org/10.17632/xpbhrtx7nd.1
Original numerical data (Quantitative RT-PCR Ct values and analyses and microscopy image quantifications)	This paper	Mendeley data https://doi.org/10.17632/xpbhrtx7nd.1
Original microscopy images	This paper	Mendeley data https://doi.org/10.68224/xpbhrtx7nd.1

**Experimental models: Cell lines**		

HEK293T/17	ATCC	Cat# CRL-11268
U-2 OS	ATCC	Cat# HTB-96
Human skin fibroblasts (control)	This paper	N017- P020 & F051-P003
Human skin fibroblasts (*UBA7* p.Lys709Serfs∗45)	This paper	P1-1312 and P2-0997
High Five cells	Thermo Fisher Scientific	Cat# B85502
Sf9 cells	Thermo Fisher Scientific	Cat# 11496015

**Oligonucleotides**		

Primers for Quantitative RT-PCR, see Table S3	This paper	N/A
Primers for PCR-cloning, see Table S3	This paper	N/A

**Recombinant DNA**		

pCAGGS-HA-UBA7	Afsar et al.^2^	N/A
pFLAG-CMV2-UBE2L6	Afsar et al.^2^	N/A
pCAGGS-V5-ISG15	Afsar et al.^2^	N/A
pcDNA3-Myc-HERC5	Afsar et al.^2^	N/A
pLIB GST-UBA7	Wallace et al.^3^	N/A
pLIB GST-UBA7 p.Val548Leu	This paper	N/A
pLIB GST-UBA7 p.Lys709Serfs∗45	This paper	N/A
pCAGGS-HA-UBA7 p.Trp311∗	This paper	Addgene # 251244
pCAGGS-HA-UBA7 p.Val548Leu	This paper	Addgene # 246473
pCAGGS-HA-UBA7 p.Lys709Serfs∗45	This paper	Addgene # 246474
pCAGGS-GFP-BamHI-IRES-mCherry	Bandi et al.^25^	Addgene #232247
pCAGGS-GFP-UBA7 WT-IRES-mCherry	This paper	Addgene # 246475
pCAGGS-GFP-UBA7 p.Trp311∗-IRES-mCherry	This paper	Addgene # 251245
pCAGGS-GFP-UBA7 p.Val548Leu -IRES-mCherry	This paper	Addgene # 246476
pCAGGS-GFP-UBA7 p.Lys709Serfs∗45-IRES-mCherry	This paper	Addgene # 246477

**Software and algorithms**		

AlphaFold 3	DeepMind & Isomorphic Labs	https://alphafoldserver.com/
PyMOL	Schrodinger	https://www.pymol.org/
ChimeraX v1.3	University of California San Francisco	https://www.cgl.ucsf.edu/chimerax/
FlowJo	BD Biosciences	https://www.flowjo.com/
Photoshop	Adobe	https://www.adobe.com/products/photoshop/
Illustrator	Adobe	https://www.adobe.com/products/illustrator/
Image Lab	BIO-RAD	https://www.bio-rad.com/en-us/product/image-lab-software
Image Studio	LICORbio	https://www.licorbio.com/image-studio
Arivis Pro	Zeiss	https://www.zeiss.com/microscopy/en/products/software/arivis-pro.html
GraphPad Prism v10.4.2	GraphPad Software Inc.	https://www.graphpad.com/
NIS-Elements	Nikon	https://www.microscope.healthcare.nikon.com/es_AMS/products/software/nis-elements
CADD (phred)	Schubach et al.^39^	https://cadd.gs.washington.edu/
SIFT	Sim et al.^40^	https://sift.bii.a-star.edu.sg/
PolyPhen-2	Adzhubei et al.^41^	http://genetics.bwh.harvard.edu/pph2/
MutationTaster	Schwarz et al.^42^	https://www.mutationtaster.org/
AlphaMissense	Cheng et al.^43^	https://github.com/google-deepmind/alphamissense
REVEL	Ioannidis et al.^44^	https://sites.google.com/site/revelgenomics/

### Experimental model and study participant details

#### Human subject recruitment and genomic investigations

Through international collaboration via our networks and GeneMatcher,^45^ we identified 4 individuals carrying biallelic *UBA7* variants. All individuals were previously investigated during routine clinical visits by their treating physicians, and genetic analyses were performed according to routine diagnostic procedures. Their demographic variables are detailed in Table 1. All investigations occurred clinically. Using genome-wide technologies for diagnostic purposes was previously approved by the Erasmus MC institutional review board (MEC-2012-387). At each recruiting site, written informed consent was obtained for all diagnostics, and written informed consent was obtained from all probands and their parents for publication of medical data, in line with the Declaration of Helsinki.

#### Human primary cells

Skin fibroblast cell lines were obtained in a clinical setting for routine clinical investigations and rest material was repurposed for research. The collection and research use of control fibroblast cell lines F051-P003 (male) and N017-P02 (female), which were derived from the consenting individuals, were conducted under written informed consent and approved by WCG IRB protocols #20120789 and #20160516, respectively. The collection and research use of patient-derived fibroblasts from individuals 1 and 2 (Fibroblast lines P1-1312 and P2-0997, both male and harboring the *UBA7* p.Lys709Serfs∗45 variant) were conducted with written informed consent from the probands and their parents, under protocol MEC-2017-341 approved by the Erasmus MC institutional review board. Primary fibroblasts were cultured in DMEM with 15% (v/v) FBS, 2 mM L-glutamine, 1 mM sodium pyruvate, and 100 U/mL Penicillin/Streptomycin. Experiments were performed with primary fibroblasts in passages 5 to 15. Fibroblast cell lines were maintained with standard growth conditions in an incubator with 5% CO_2_ and 37 °C.

#### Human cell lines

HEK293T/17 (female, ATCC CRL-11268) and U-2 OS (female, ATCC HTB-96) cell lines were cultured in Dulbecco’s Modified Eagle Medium (DMEM) supplemented with 10% (v/v) Fetal Bovine Serum (FBS), 2 mM L-glutamine, and 100 U/mL Penicillin/Streptomycin. Experiments were performed with cells in passages 5 to 15. Cell lines were maintained with standard growth conditions in an incubator with 5% CO_2_ and 37 °C. HEK293T/17 cells were transfected with PEI MAX Linear Polyethylenimine Hydrochloride reagent (Polysciences), and U-2 OS cells were transfected with Lipofectamine LTX Reagent with PLUS (Thermo Fisher Scientific), according to the manufacturer’s instructions. HEK293T/17 and U-2 OS cells were obtained directly from ATCC which authenticates and performs STR profiling on all distribution lots. The authors did not conduct independent STR authentication. These cell lines were routinely tested for mycoplasma contamination using quantitative RT-PCR including positive and negative controls, and only mycoplasma-negative cells were used for experiments.

#### Insect cells

High Five cells were grown in ESF 921 media (Oxford expression technologies #500304) and Sf9 cells were grown in Sf-900 II SFM media (Thermo Fisher Scientific #10902096). High Five cells were grown in suspension and Sf9 cells were grown in suspension or adherent to plates at 27 °C in a non-CO_2_, non-humidified incubator. Suspension cultures were grown with gentle shaking at 120 rpm.

#### Bacterial cells

DH10EMBacY, BL21-CodonPlus (DE3)-RIPL, Stellar Competent Cells (HST08), and NEB 5-alpha Competent (DH5α) *E. coli* strains were grown in LB media or LB agar at 37 °C in non-CO_2_, non-humidified shakers or incubators.

### Method details

#### Protein structure *in silico* predictions

The wild-type UBA7 (UniProt accession: P41226) model was retrieved from the AlphaFold 2 Protein Structure Database^46^ and UBA7 variants (p.Trp311∗, pLys709Serfs∗45) were generated using AlphaFold 3.^47^ Modeling of the p.Val548Leu variant was based on the cryo-EM structure of UBA7 bound to ISG15 and UBE2L6 (PDB: 8OIF) and performed using the mutagenesis feature of PyMOL. UBA7 domain boundaries were colored as previously described^3^ and structural figures were generated using PyMol and ChimeraX^48^ version 1.3.

#### Gene cloning

pCAGGS-HA-UBA7, pFLAG-CMV2-UBE2L6, pCAGGS-V5-ISG15 and pcDNA3-Myc-HERC5^2^ were a kind gift from Dr. Michaela U. Gack (Florida Research and Innovation Center). The UBA7 variants p.Trp311∗, p.Val548Leu and p.Lys709Serfs∗45 (exact nucleotide sequences found in the patients) were introduced into pCAGGS-HA-UBA7 via PCR and In-Fusion cloning. UBA7 wild type, p.Trp311∗, p.Val548Leu, and p.Lys709Serfs∗45 were cloned into the pCAGGS-GFP-BamHI-IRES-mCherry vector^25^ (Addgene 232247) immediately after the GFP in a BamHI site. Baculovirus constructs for recombinant expression of UBA7 variants in insect cells were created by introducing UBA7 WT, p.Val548Leu and p.Lys709Serfs∗45 into the pLIB vector as N-terminal GST fusion with a TEV cleavage site. All clones were further confirmed by DNA sequencing (Plasmidsaurus). Primer sequences used for cloning are listed in Table S3.

#### In-cell ISGylation reconstitution assay

HEK293T/17 cells were transfected with ISGylation enzyme expression plasmids pCAGGS-HA-UBA7, pFLAG-CMV2-UBE2L6, pCAGGS-V5-ISG15 and pcDNA3-Myc-HERC5 at a ratio of 1(E1):0.25(E2):1(E3):0.25(ISG15) with 1 mg/mL PEI MAX Linear Polyethylenimine Hydrochloride reagent (Polysciences), and cells were lysed 48 h after transfection.

#### Pharmacological treatments

Fibroblast cell lines were treated with 1 μM MG132 for 24 h, and DMSO served as a control. For ISGylation experiments, fibroblast cell lines were treated with the 50 pM and 100 pM interferon beta (IFNβ) for 48 h, and 0.1% BSA served as a control.

#### Immunoblotting

Cells were harvested in IP-MS buffer (20 mM Tris (pH 7.4), 150 mM NaCl, 1 mM EDTA, 1% Nonidet P-40 (NP-40) (v/v), 0.5% sodium deoxycholate (w/v), 10 mM b-glycerophosphate, 10 mM sodium pyrophosphate, 1 mM NaF, 2 mM Na3VO4, and cOmplete protease inhibitor cocktail tablets (MilliporeSigma)). Protein concentration was measured with BCA Protein assay (Thermo Fisher Scientific) and protein samples were separated in NuPAGE 4–12% Bis-Tris gradient gels (Thermo Fisher Scientific) before proteins were transferred into PVDF or Nitrocellulose membranes that were stained with Ponceau S Staining Solution (Millipore Sigma). Membranes were blocked with 5% (w/v) nonfat milk Tris buffer saline buffer with Tween 20 (TBS-T) and incubated with primary antibodies (See key resources table). HRP-conjugated secondary antibodies were used for electrochemiluminescence detection using a ChemiDoc MP (BIO-RAD) instrument or IRDye 800CW or 680RD-conjugated antibodies were used for fluorescence detection using an Odyssey FC Imager (LI-COR Biotech). Figures were assembled using Image Lab (BIO-RAD), Image Studio (LI-COR Biotech) and Illustrator (Adobe).

#### Protein purification and fluorescent labeling

Proteins were purified as described previously.^3^ Briefly, ISG15 C0 C78S and UBE2L6 were expressed in *Escherichia coli* BL21 cells and purified using a tandem nickel and glutathione (ISG15 C0 C78S) or a single nickel (UBE2L6) affinity purification. UBA7 was expressed in High Five cells (Thermo Fisher #B85502) infected with baculovirus prepared using Sf9 cells (Thermo Fisher #11496015). UBA7 and UBA7 variants were purified using glutathione affinity resin. Affinity tags were cleaved overnight at 4 °C using TEV, SENP1, or 3C proteases. After cleavage, proteins were further purified using ion exchange and size-exclusion chromatography as needed. ISG15 fluorescent labeling with BODIPY fluorescent maleimide (Thermo Fisher) was performed as described previously.^3^

#### E1 and E2 charging assays

For E1 charging assays, 1 μM UBA7 was incubated with 125 nM ISG15 at 25°C in reaction buffer (50 mM Tris pH 7.5, 150 mM NaCl, 10 mM MgCl2, 10 mM ATP). Reactions were stopped at the indicated timepoints by the addition of LDS. Reactions were separated by SDS-PAGE and visualised using an Amersham Typhoon fluorescent imager.

For E2 charging assays, UBA7∼ISG15 complexes were formed by incubating 2.5 μM UBA7 with 3 μM fluorescent ISG15 in charging buffer (50 mM HEPES pH 7.5, 150 mM NaCl, 5 mM MgCl_2_, 5 mM ATP) for 15 min at 25 °C. UBA7∼ISG15 charging reactions were stopped by diluting the sample 10-fold in quenching buffer (50 mM HEPES pH 7.5, 150 mM NaCl, 100 mM EDTA). UBE2L6 (2 μM) was then added to the preformed E1 thioester complexes. Reactions were stopped at the indicated timepoints by the addition of LDS. Reactions were separated by SDS-PAGE and visualised using an Amersham Typhoon fluorescent imager.

#### Protein stability reporter assays

For protein stability analysis, HEK293T/17 cells were seeded into 6-well plates, and 500 ng DNA of pCAGGS-GFP-IRES-mCherry, pCAGGS-GFP-UBA7-WT-IRES-mCherry, pCAGGS-GFP-UBA7-p.Trp311∗-IRES-mCherry, pCAGGS-GFP-UBA7-p.Val548Leu-IRES-mCherry, and pCAGGS-GFP-UBA7-p.Lys709Serfs∗45-IRES-mCherry, were transfected. After 24 h of transfection, cells were harvested by trypsinization, and cells were resuspended in 200 μL of PBS and analyzed in a Fortessa analyzer (BD). Data were analyzed using the FlowJo software (BD), and the median values for the GFP and mCherry fluorescence for each sample were calculated and histograms were plotted.

#### Microscopy

For localization studies, U-2 OS cells were seeded into glass coverslips, and 500 ng DNA of pCAGGS-GFP-IRES-mCherry, pCAGGS-GFP-UBA7-WT-IRES-mCherry, pCAGGS-GFP-UBA7-p.Trp311∗-IRES-mCherry, pCAGGS-GFP-UBA7-p.Val548Leu-IRES-mCherry, and pCAGGS-GFP-UBA7-p.Lys709Serfs∗45-IRES-mCherry, were transfected. Cells were fixed with 4% paraformaldehyde (w/v) in PBS. Fixed cells were stained with Hoechst to mark the DNA. Cells were mounted in glass slides using FluorSave (MilliporeSigma). Super resolution confocal images were acquired with a Nikon CSU-W1 SoRa spinning disc confocal microscope using the NIS-Elements software. Images were assembled using Photoshop and Illustrator (Adobe).

#### Quantitative RT-PCR

Human control and patient fibroblast cells were harvested in TRIzol reagent (Thermo Fisher Scientific), and RNA was extracted using the phenol chloroform method. 1 μg of total RNA was converted into cDNA using the qScript cDNA Synthesis Kit (Quanta Bio). Quantitative RT-PCR reactions were prepared using TB Green Premix Ex Taq (Takara Bio) and were analyzed in a CFX384 real-time PCR system (BIO-RAD). RNA expression was estimated via the ΔΔCt method and normalized to *GAPDH* expression. Primers used are listed in Table S3.

### Quantification and statistical analysis

Quantification of the nuclear versus cytoplasmic UBA7 signal in microscopy images was performed using Arivis Pro (Zeiss). Numerical data were analyzed in Excel (Microsoft) and statistical analyses were performed in GraphPad Prism software (v10.4.2 GraphPad Software Inc.). Numerical data are represented as box-and-whiskers plots displaying all points. Box spans from the first to the third quartile. Whiskers extend from the minimum to the maximum values. Experiments were performed in at least three biological replicates unless otherwise indicated and figures display representative experiments. Statistical significance was estimated using One-way ANOVA followed by Tukey’s post hoc test. Significance was defined as *p* < 0.05.
