# Supplementary material for: ISGylation is disrupted by UBA7 gene variants identified in individuals with neurodevelopmental disorder phenotypes
Source: iScience. 2026 Mar 30;29(5):115454. doi: 10.1016/j.isci.2026.115454 (PMC13097003; doi:10.1016/j.isci.2026.115454)
Supplement: Document S1. Figures S1, S2, Tables S1–S3, and Data S1 [file mmc1.pdf]

iScience, Volume *29*

Supplemental information

ISGylation is disrupted by *UBA7* gene variants identified in individuals with neurodevelopmental disorder phenotypes

Venkateshwarlu Bandi, Myrrhe Venema, Iona Wallace, Merel O. Mol, Anita Nikoncuk, Rachel Schot, Marjon van Slegtenhorst, Emilia K. Bijlsma, Amjad Khan, Susan M. White, Rocio Rius, Martin B. Delatycki, Vinodh Narayanan, Kirby N. Swatek, Tahsin Stefan Barakat, and Francisco Bustos

Table S1. Additional variants found in genetic investigations (Related to Table 1).

Individual	Other variants found	ClinVar classification variant	Associated OMIM phenotype
1	NM_014981.1(MYH15):c.3415A>G, p.(Thr1139Ala), homozygous	No ClinVar match	No associated OMIM phenotype
1	NM_001142503.2(STARD8):c.280C>A, p.(His94Asn), maternal	VUS[a]	No associated OMIM phenotype
2		Benign/VUS	X-linked susceptibility to autism-4, OMIM #300830
3	N/A	N/A	N/A
4	NM_052859.4(RFT1):c.109C>T; p.Arg37Cys, homozygous	VUS	Congenital disorder of glycosylation type In, OMIM #612015

[a] Variant of uncertain significance

Table S2. Analysis of variants identified in individual 4 (Related to Table 1).

Genetic analysis	UBA7 Val548Leu	RFT1 Arg37Cys
Genomic position variant	chr3:g.49810175C>A	chr3:g.53125949G>A
Transcript variant	NM_003335.3:c.1642G>T	NM_052859.4:c.109C>T
Protein variant	p.Val548Leu	p.Arg37Cys
Zygosity	Homozygous	Homozygous
Functional analysis variant	No significant impairment of enzymatic function	N/A
GnomAD v4.1.0		
Allele number	1613206	1461496
Allele count	104	7
Allele frequency	0.00006447	0.000004790
Number of homozygotes	1	0
GnomAD v4.1.0 - non-UKB		
Allele number	780120	628426
Allele count	67	4
Allele frequency	0.00008588	0.000006365
Number of homozygotes	0	0
Variant predictions		
CADD (phred)	12.11	34.00
SIFT	0.049 (damaging)	0.0 (damaging)
PolyPhen-2 HumDiv	0.04 (benign)	1.0 (probably damaging)
PolyPhen-2 HumVar	0.119 (benign)	0.998 (probably damaging)
MutationTaster	Disease causing, probability score 0.95796	Disease causing, probability score 0.999999999999911
AlphaMissense	0.159 (likely benign)	0.396 (ambiguous)
REVEL	0.104 (benign)	0.954 (damaging)

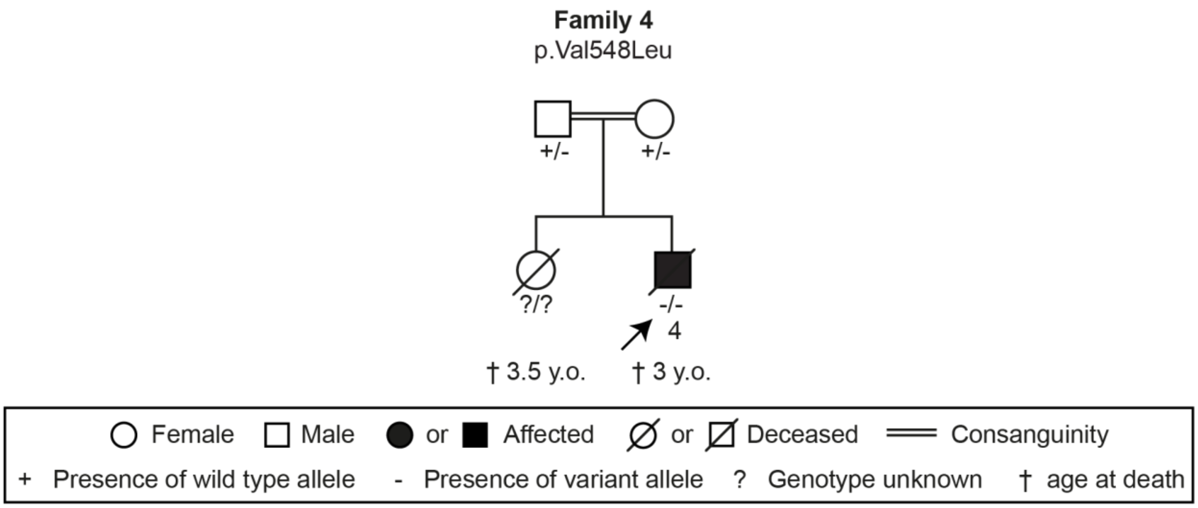

Figure S1. Family segregation pattern of the *UBA7* p.Val548Leu variant (Related to Figure 1A). Pedigree of family 4, indicating affected individual 4 with an arrow. A legend is provided for the symbols used. Age at death in years is abbreviated as y.o.

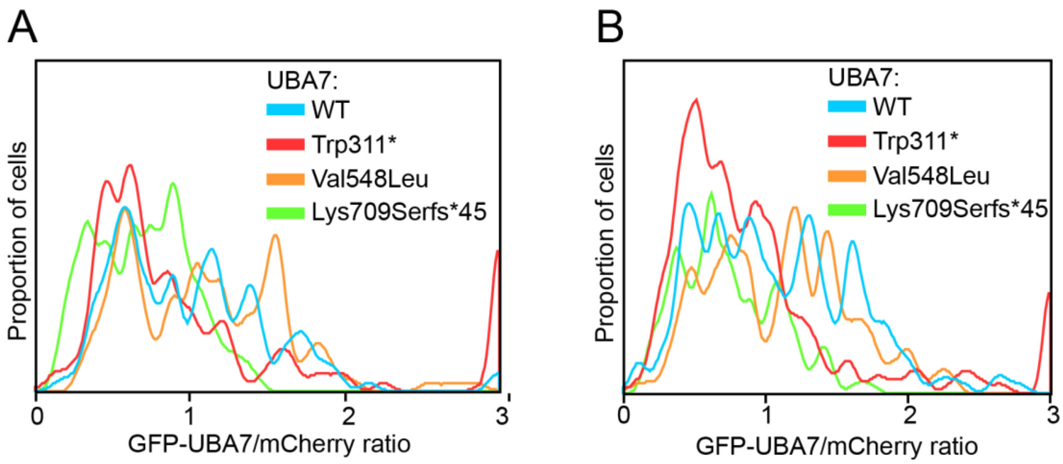

Figure S2. UBA7 Trp311* and Lys709Serfs*45 mutant proteins display reduced stability (Related to Figure 3A). Flow cytometry histogram representation of replicate 2 (A) and 3 (B) of the UBA7 protein stability reporter assay shown in figure 3A. HEK293T/17 cells were transfected with GFP-IRES-mCherry plasmids encoding UBA7 wild type (WT), p.Trp311*, Val548Leu and Lys709Serfs*45 vectors and the ratio of GFP versus mCherry fluorescence was determined.

Table S3: Oligonucleotides used in this paper (Related to STAR Methods).

**Quantitative RT-PCR primers**	
**Name**	**Sequence (5'-3')**
UBA7 Forward	GAACTCTTCCGACTGTCTGCA
UBA7 Reverse	AGTCTTGCCAGTTCTGTGGACG
GAPDH Forward	AGA AGGCTGGGGCTCATTTG
GAPDH Reverse	AGGGGCCATCCACAGTCTTC
**PCR-cloning primers**	
**Name**	**Sequence (5'-3')**
pLIB UBA7 p.Val548Leu mutagenesis Forward	CGCTATTTGGCTGCTCGTTGCACCCACTATCTG
pLIB UBA7 p.Val548Leu mutagenesis Reverse	GAGCAGCCAAATAGCGCCGGGCCTGGAAACTGTC
pLIB linearisation Forward	TAAGATAAAAGCTTGTCGAGAAGTACTAGAGGATCATAATCAGCC
pLIB linearisation Reverse	GGATCCCTGAAAATACAGGTTTTC
UBA7 p.Lys709Serfs*45 to pLIB Forward	TATTTTCAGGGATCCATGGATGCCCTGGACGCTTCGAAGC
UBA7 p.Lys709Serfs*45 to pLIB Reverse	AAGCTTTTATCTTATCAGCAGCTCCCTGAGTGCAGTCCAG
pCAGGS-HA-UBA7 p.Trp311* Mutagenesis Forward	CAGCCCTGAGATCCTGTTGATGCAGAGACTGTGG
pCAGGS-HA-UBA7 p.Trp311* Mutagenesis Reverse	AGGATCTCAGGGCTGGGGTGGCCGG
pCAGGS-HA-UBA7 p.Val548Leu Mutagenesis Forward	GGCGCTATTTGGCTGCTCGTTGCACCC
pCAGGS-HA-UBA7 p.Val548Leu Mutagenesis Reverse	CAGCCAAATAGCGCCGGGCCTGGAAAC
pCAGGS-HA-UBA7 p.Lys709Serfs*45 Mutagenesis Forward	AGGTCCCAGTGTCCCCAGCCCTTGGA
pCAGGS-HA-UBA7 p.Lys709Serfs*45 Mutagenesis Reverse	GGGACACTGGGACCTGACCAGAAGGGA
UBA7 to pCAGGS-GFP-BamHI-IRES-mCherry Forward	AGGCGGAGGCGGATCCATGGATGCCCTGGACGCTTC
UBA7 (WT and p.Val548Leu) to pCAGGS-GFP-BamHI-IRES-mCherry Reverse	AGAGGGGCTAGGATCCTCACAGCTCATAGTGCAGAGG
UBA7 (p.Trp311*) to pCAGGS-GFP-BamHI-IRES-mCherry Reverse	AGAGGGGCTAGGATCCTCACAGCTCATAGTGCAGAGG
UBA7 (p.Lys709Serfs*45) to pCAGGS-GFP-BamHI-IRES-mCherry Reverse	AGAGGGGCTAGGATCCTCAGCAGCTCCCTGAGTGC

Data S1: Case report individual 4.

Individual 4 is a male of Afghani descent. He was born to consanguineous parents (first cousins) at term, and had a sister who could not be investigated, and who was similarly affected, with no profound developmental delay, who died at 3.5 years of age. He presented at the age of 3 months with severe intractable epilepsy and developmental delay. During the course of his life, he had profound developmental delay, with regression of skills. Further neurological symptoms included axial hypotonia, spasticity, nystagmus and bilateral auditory neuropathy. He made no visual interaction.

He suffered from epileptic seizures, mainly asymmetric tonic with later clonic jerking, though occasional tonic seizures were also noted. The frequency of seizures was severe, with multiple seizures hourly and over 50 seizures per day. He was treated with levetiracetam with no reported benefit, and valproate, which reportedly worsened the seizures. Brain MRI was performed at 7 months of age and showed a thin corpus callosum with reduced white matter and cortical atrophy. No dysmorphic features were noted, though he was microcephalic, with a head circumference below the third percentile. He died at the age of 3 years. His clinical phenotype is considered much more severe and shows very little phenotypic overlap to individual 1-3.

He was screened for metabolic diseases, including lactate, urine amino acids, organic acids and glycosaminoglycans, as well as transferrin isoforms, which were all normal. Chromosomal microarray showed no abnormalities. Whole exome sequencing revealed homozygosity for two missense variants of unknown clinical significance in *UBA7* (NM_003335.3:c.1642G>T; p.Val548Leu) and in *RFT1* (NM_052859.4:c.109C>T; p.Arg37Cys). Characteristics of these variants are summarized in **Table S2** and include a CADD score of 12.11 for the *UBA7* variant and 34.00 for the *RFT1* variant.

While for *UBA7* no previous disease-gene association has been described, deficiencies in *RFT1* cause a congenital disorder of glycosylation syndrome (CDG). RFT1-CDG is associated with developmental delay, epilepsy, hypotonia and sensorineural deafness, which would fit the patient's phenotype very well [S1]. The established laboratory test for CDG syndromes is analysis of transferrin isoforms, which were normal in this patient, which would suggest that the missense variant identified in the individual might not be causative [S2]. However, multiple cases in literature have been described of RFT1-CDG with normal transferrin isoform patterns, indicating that normal analysis of transferrins does not necessarily exclude causality [S3,4].

The *UBA7* p.Val548Leu variant shows variable prediction scores (**Table S2**). It was therefore also tested functionally *in vitro* in this paper and showed no difference in enzymatic activity compared to wild type. The *RFT1*-Arg37Cys was not tested functionally but does show more severe prediction scores. Additionally, the *UBA7* p.Val548Leu variant was present once in homozygous state in gnomAD v4.1.0, whereas *RFT1*-Arg37Cys was never found in a homozygous state. While some of the biobanks included in gnomAD v4.1.0 do not have a phenotypic restriction, and therefore the presence of homozygotes in gnomAD does not automatically rule out whether a variant may cause disease or not, this seems more likely for diseases with a variable or milder phenotype [S5]. The phenotype of this patient was very severe, which makes presence of homozygotes in gnomAD a more substantial argument against the *UBA7* p.Val548Leu variant being causative of the patient's phenotype in this case.

Taking all the information of this case together, it seems unlikely that the *UBA7* p.Val548Leu variant is the main cause of this patient's phenotype, with the variant in *RFT1* being a more likely candidate. However, we are also not yet able to completely rule out whether the *UBA7* variant might have had an additional contributory effect to the phenotype.

Supplemental references

S1. Vleugels, W., Haeuptle, M.A., Ng, B.G., Michalski, J.C., Battini, R., Dionisi-Vici, C., Ludman, M.D., Jaeken, J., Foulquier, F., Freeze, H.H., et al. (2009). RFT1 deficiency in three novel CDG patients. Hum Mutat 30, 1428-1434. 10.1002/humu.21085.

S2. Stibler, H., Holzbach, U., and Kristiansson, B. (1998). Isoforms and levels of transferrin, antithrombin, alpha(1)-antitrypsin and thyroxine-binding globulin in 48 patients with carbohydrate-deficient glycoprotein syndrome type I. Scand J Clin Lab Invest 58, 55-61. 10.1080/00365519850186832.

S3. Abiramalatha, T., Arunachal, G., Muthusamy, K., and Thomas, N. (2019). A family with floppy neonates with severe respiratory insufficiency: A lethal phenotype of RFT1-CDG due to a novel mutation. Eur J Med Genet 62, 248-253. 10.1016/j.ejmg.2018.07.023.

S4. Aeby, A., Prigogine, C., Vilain, C., Malfilatre, G., Jaeken, J., Lederer, D., and Van Bogaert, P. (2016). RFT1-congenital disorder of glycosylation (CDG) syndrome: a cause of early-onset severe epilepsy. Epileptic Disord 18, 92-96. 10.1684/epd.2016.0802.

S5. Baxter, S., Singer-Berk, M., Russell, K., Amin, M., Glaze, C., Grant, R., Lee, J., Watts, N., Wilson, M., Rehm, H., and O'Donnell-Luria, A. (2024). P138: Evaluating the impact of gnomAD v4 on genetic prevalence estimates*. Genetics in Medicine Open 2, 101035. 10.1016/j.gimo.2024.101035.
